# Fostering the Quality of Care for People with Chronic Diseases, from Theory to Practice: The Development of Good Practices in Disease Prevention and Care in JA CHRODIS PLUS Using JA CHRODIS Recommendations and Quality Criteria

**DOI:** 10.3390/ijerph17030951

**Published:** 2020-02-04

**Authors:** Jelka Zaletel, Marina Maggini

**Affiliations:** 1 National Institute of Public Health Slovenia, Trubarjeva 2, 1000 Ljubljana, Slovenia; 2 National Institute of Health, Viale Regina Elena 299, 00161 Rome, Italy; marina.maggini@iss.it

**Keywords:** chronic diseases, quality of care, meaningful patient involvement, co-design of practices, sustainability, scalability

## Abstract

In the frame of joint action in chronic diseases (JA CHRODIS), an extensive process at the European Union level was carried out to identify a core set of quality criteria and to formulate recommendations that improved prevention, early detection, and quality of care for people with chronic diseases. Diabetes was used as a model disease. The core set of quality criteria may be applied to develop and improve practices, programs, strategies, and policies in various domains (e.g., prevention, care, health promotion, education, and training). The quality criteria are general enough to be applied in countries with different political, administrative, social, and health care organizations. Moreover, they can be applied to a number of other chronic diseases. JA CHRODIS recommendations and quality criteria are being tested in a series of pilot actions within the JA CHRODIS PLUS. A total of 15 partners representing nine European countries worked together to implement pilot actions and generate practical lessons that could contribute to the further uptake and use of JA CHRODIS recommendations. Special emphasis is given to meaningful patient involvement in co-designing the pilot actions and to the sustainability and scalability of the pilot actions. These insights were found to be at the core of the learning from pilot actions to foster high quality care for people with chronic diseases.

## 1. Introduction

Redesigning health care systems to better meet the complex needs of persons with one or multiple chronic diseases is a challenge for decision-makers and leaders in health care all over Europe. In 2011, the General Assembly of the United Nations [1], with support from the European Union (EU), adopted a political declaration on the prevention and control of non-communicable diseases. The European Summit on chronic diseases (Brussels, 2014) stressed the need for joint efforts at the European level to optimize resources and sustained commitment to address major chronic diseases. They acknowledged the need for a coalition across society to prevent chronic diseases, preserving the best state of health and sustainability of a modern health system, with the aim of maximizing a healthy life for European citizens [2]. In line with that decision, the European joint action on chronic diseases and promoting healthy ageing across the life cycle (JA CHRODIS 2014–2017) [3] offered results that addressed health promotion, prevention of chronic diseases, multimorbidity, and recommendations for implementing good practices (i.e., interventions, policies, strategies, programs, and/or practices) to improve prevention and quality of care for people with (one or several) chronic diseases. Diabetes was used as a model disease. The recommendations and the corresponding set of quality criteria can be applied to various domains (prevention, care, health promotion, education, and training), and in countries with different political, administrative, social, and health care organizations. In JA CHRODIS PLUS, which implements good practices for chronic disease [4], the goal is to support member states through cross-national initiatives identified in JA CHRODIS, such as JA CHRODIS recommendations and quality criteria, with and overall aim to reduce the burden of chronic diseases, while also assuring health systems are sustainable and responsive. In JA CHRODIS PLUS, partners from Finland, Slovenia, Croatia, Serbia, Greece, Germany, and Bulgaria agreed to evaluate applicability of the JA CHRODIS recommendations and quality criteria through pilot actions in different settings, domains, and health care organizations. The aim is to evaluate their transferability, to identify key enablers, and barriers for their use and to develop a guide for future use. 

This paper aims to present JA CHRODIS recommendations and quality criteria, along with the method used for their development, and to present ongoing implementation activities. 

## 2. Materials and Methods

### 2.1. JA CHRODIS Recommendations and Quality Criteria

In JA CHRODIS, there is a work package for diabetes as a model complex chronic disease focused on health promotion, prevention for those at risk, comprehensive multifactorial and interdisciplinary care, educational strategies for people with diabetes, training for health professionals, and development and implementation of national diabetes policies. The objective was to define recommendations and quality criteria for developing a good practice that assesses the practice related to any chronic disease. The “practice” may be an intervention, policy, strategy, program, process, or (clinical) practice. Recommendations and quality criteria could be applied to various domains of prevention, care, health promotion, education, and training, especially in countries with different political, administrative, social, and health care organizations. 

First, the partners conducted a survey to provide a structured overview of current practices (interventions, initiatives, approaches, or equivalents) [5], followed by a SWOT analysis by country. The aim was to offer insights and partners’ point of view on what makes a policy/program applicable, sustainable, and effective from a public health and stakeholder perspectives. Further, we wanted to consider the necessary preconditions for its implementation and lessons learnt from the experience. It also provided a contextual perspective of the setting where good practices are developed. 

In parallel, a literature review was conducted to identify quality criteria for practices on health promotion, diabetes prevention targeted at people at risk, management of care, patients’ education, and health professionals’ training [6,7,8,9,10].

The results of the survey, SWOTs per country, and literature review findings were used by an expert group to define a list of universal quality criteria to be used in the RAND (Research And Development project) modified DELPHI process [11]. Criteria were mapped out and redundancies were collapsed or rephrased. The resulting criteria were organized into 10 thematic drivers, including a total of 71 items clustered. Further, it included the first online questionnaire submitted to an expert panel. This expert panel decided on the suitability and priority of a series of criteria to assess whether a practice can be regarded as ‘good practice’ in the field of prevention and care of type 2 diabetes. A total of 28 European experts (i.e., diabetologists/endocrinologists, general practitioners, nurses, representative of patients and governmental bodies, public health professionals, and researchers) were invited to join the panel. Participants came from different countries (i.e., Austria, Belgium, Finland, France, Germany, Greece, Ireland, Italy, Norway, Portugal, Romania, Slovenia, Spain, and United Kingdom), covering a variety of health system models. Consultation with the expert panel followed the RAND modified DELPHI methodology. In short, RAND modified DELPHI methodology, which entailed two online rounds using a web-based questionnaire, followed by a face-to-face meeting. The consultation was launched in April 2016. Panel experts completed the questionnaire in the first round; 26 completed it during the second round. In both the first and second rounds, experts were invited to add any driver they thought relevant or missing. No additional items were suggested during the process. The expert meeting refined and prioritized criteria and was held in Brussels on 12–13 May 2016. In total, 16 experts were able to attend the meeting. After the definition of the final set of criteria, experts weighed criteria by distributing 100 points among them (criteria weight) and weighed categories for each criterion (category weight). A trained facilitator following a structured consensus methodology conducted the face-to-face meeting.

### 2.2. Pilot Actions

The partners agreed on a methodology to design the pilot action plan based on JA CHRODIS recommendations, which supported and monitored the implementation of the activities, including a plan-do-study-act (PDSA) cycle and the final reporting based on JA CHRODIS quality criteria. The design, implementation, and evaluation of pilot actions involved the following steps:

1. Establishment of the core management group and the implementation group that design, implement, and monitor the pilot action.
2. Definition of the scope of the pilot action.
3. Analyzing the situation and context using SWOT methodology.
4. Designing the pilot action plan.
5. Implementing the activities as defined in the pilot action plan.
6. Monitoring and adapting the activities based on the principles of PDSA cycles.
7. Reporting and sharing knowledge of the results and experience using SQUIRE 2.0 [12]. Guidelines as framework for reporting.

The process of implementation is being supported by:

- regular contacts and sharing the experiences, including identified barriers and obstacles;
- templates, developed together with partners with pilot actions; and
- face-to-face workshops: general implementation strategy workshop (February 2018), workshop to develop pilot action plan (June 2018), workshop to support interim follow-up (December 2018), that was enhanced by the study visits at pilot action implementation sites (March–April 2019), and workshop to finalize the individual pilot action report (October 2019).

If the pilot action needed ethical approval, the national legislation was strictly followed. The subjects gave their informed consent for inclusion before they participated in the pilot action. If the ethical approval was needed, the pilot action was conducted in accordance with the Declaration of Helsinki and the protocol was approved by the respective Ethics Committee.

## 3. Results

Below is a summary of the results of the structured survey of the counties: 19 countries, with 63 experts, contributed to the collection of data. In short, the importance of the prevention of diabetes is acknowledged and addressed at the policy level: 75% of countries report that diabetes prevention is supported by national policies and legislation. However, early identification of people at risk is only supported by 63.2%. This might indicate that the prevention of diabetes is recognized at the population level (e.g., advocating physical activity and healthy body weight as means to prevent diabetes), but specific actions targeted to people at risk are not addressed in diabetes policies in all countries. As such, 18 out of the 19 respondents have a management program for diabetes, but only 50% of the programs take into consideration vulnerable groups, e.g., ethnic minorities and lower socioeconomic groups. Defined care pathways exist to deal with persons with diabetes, either with or at risk for micro- and macrovascular complications, in 77.8% of the countries. Most of the programs (72.2%) are monitored through intermediate outcome indicators and 44.4% use long-term outcome indicators, but 16.7% of the countries did not use any kind of indicator. Further, 15 out of the 19 participating countries reported educational programs for persons with diabetes. The core criteria of the quality of education programs are defined, e.g., the goal, the rationale, the target group, the setting, and the scheduling of the education sessions. More than half reported to have an evidence-based curriculum and defined specific education methods and didactics. However, only 60% of the participants reported that the curriculum is evaluated and 20% reported that long-term effect indicators were used. Training programs for professionals exist in 12 of the 19 participating countries and the core criteria of the quality of training programs appear to be defined. More than half reported to have an evidence-based curriculum and specific training methods and didactics. As for the education program, a low number (38.5%) reported that a monitoring of effectiveness and quality of the training program is defined. Further, 30.8% reported that intermediate outcome indicators are applied.

Below is a summary for the results of the SWOT analyses by country [13]. A total of 53 stakeholders in 12 countries contributed to the SWOT reporting and analyzing 39 policies, programs, projects, and interventions. According to the responders, to be a “success” a policy or a program needs to be dynamic, bottom up, flexible, integrated, multi-intersectoral, and equity oriented. External communication and dissemination are key points for success, and the partnership among stakeholders should be kept engaged throughout the process, as a strong scientific background is considered a key point. Strategies should be comprehensive and address the most common risk factors of the main non-communicable chronic diseases. A clear description of care pathways is needed, supported by an information system at a national, subnational, and local level. Planning and definition of sound objectives on integrated care is a leading starting point. Good educational models and care strategies are essential and need to be shared with persons with diabetes. Regular monitoring and evaluation with a defined and shared set of outcomes and indicators are identified as important drivers for program implementation. A strong and efficient leadership is needed. Some threats may stand in the way of program implementation. 

Despite improving, the culture of disease prevention and health promotion is still weak. On the other hand, from the science perspective, we still have general gaps in our knowledge of diabetes and NCDs. The prevalence of NCDs and obesity in children is growing, with the persistence of social inequalities in health. Specific legislation promoting healthy lifestyles are scarce across Europe and industry and economic lobbies in general may adversely affect political decisions and do not always support healthy lifestyle. Different care paradigms coexist and sometimes conflict with one another. Prevention and care are still seen as “competitors” for resources, workforce, and facilities, as programs and projects may compete over the same funding and same personnel. Despite some successful experiences, not one of the countries that participated reported a systematic integration of national policies or programs embracing different sectors. Moreover, university curricula and health professionals’ pre-service education are still not dealing with the changing needs of the ageing population. An opportunity that may facilitate implementation of policies/programs is the increasing awareness across European institutions and health care systems that action must be taken to address chronic conditions prevention and health promotion. Sharing and exchange of best practices of chronic care management and integrated care at the European level also acts as a motivator. some programs have been used as a model outside the original country of implementation. 

In the following subsections, JA CHRODIS recommendations defined by the RAND-modified DELPHI process is provided.

### 3.1. Design of the Practice

The design should clearly specify aims, objectives, and methods, and rely upon relevant data, theory, context, evidence, and previous practices including pilot studies. The structure, organization, and content of the practice is defined and established together with the clearly described target population (i.e., exclusion and inclusion criteria and the estimated number of participants). 

Human and material resources should be adequately estimated in relation with committed tasks. Relevant dimensions of equity have to be adequately taken into consideration and targeted. 

### 3.2. Promote the Empowerment of the Target Population

The practice should actively promote the empowerment of the target population by using appropriate mechanisms, such as self-management support, shared decision-making, education-information, value clarification, active participation in the planning process, active participation in professional training, and considering stakeholder needs in terms of enhancing/acquiring the right skills, knowledge, and behavior. 

### 3.3. Define an Evaluation and Monitoring Plan

The evaluation outcomes should be linked to action to foster continuous learning and/or improvement, and/or to reshape the practice. Evaluation and monitoring outcomes should be shared among relevant stakeholders and linked to the stated goals and objectives, taking into account social and economic aspects from both the target population and formal and informal caregiver perspectives.

### 3.4. Comprehensiveness of the Practice

The practice should consider relevant evidence on effectiveness, cost-effectiveness, quality, safety, the main contextual indicators, and underlying risks of the target population using validated tools to individual risk assessment.

### 3.5. Include Education and Training

The practice should include educational elements to promote the empowerment of the target population (e.g., strengthen their health literacy, self-management, stress management, etc.). Relevant professionals and experts are trained to support target population empowerment, and trainers/educators are qualified in terms of knowledge, techniques, and approaches.

### 3.6. Ethical Considerations

The practice should be implemented equitably (i.e., proportional to needs). The objectives and strategy are transparent to the target population and stakeholders involved. Potential burdens (i.e., psychosocial, affordability, accessibility, etc.) should be addressed to achieve a balance between benefit and burden.

The target population has the right to be informed, to decide about their care, and participation. Their right to confidentiality should be respected and enhanced.

### 3.7. Governance Approach

The practice should include organizational elements, identifying the necessary actions to remove legal, managerial, financial, or skill barriers, with the contribution of the target population, caregivers, and professionals planned for, supported, and resourced. There is a defined strategy to align staff incentives and motivation with the practice objectives.

The practice should offer a model of efficient leadership and should create ownership among the target population and several stakeholders considering multidisciplinary, multi-/intersectoral, partnerships and alliances, if appropriate. 

The best evidence and documentation supporting the practice (guidelines, protocols, etc.) should be easily available for relevant stakeholders (e.g., professionals and target populations), which should support the multidisciplinary approach for practices. 

The practice should be supported by different information and communication technologies (e.g., medical record system, dedicated software supporting the implementation of screening, social media etc.), defining a policy to ensure acceptability of information technologies among users (professionals and target population) to enable their involvement in the process of change.

### 3.8. Interaction with Regular and Relevant Systems

The practice should be integrated or interactive with regular healthcare and/or further relevant systems, enabling effective linkages between all relevant decision makers and stakeholders, and enhancing and supporting the target populations’ ability to effectively interact with the regular relevant systems.

### 3.9. Sustainability and Scalability 

The continuation of the practice should be ensured through institutional anchoring and/or ownership by the relevant stakeholders or communities, as well as supported by those who implemented it.

The sustainability strategy should consider a range of contextual factors (e.g., health and social policies, sex and gender issues, innovation, cultural trends, general economy, and epidemiological trends) that assesses the potential impact on the population targeted.

Based on the JA CHRODIS recommendations, quality criteria were refined and ranked (Table 1).

JA CHRODIS recommendations and quality criteria are used to develop, implement, monitor, and evaluate pilot actions in several EU countries. In JA CHRODIS PLUS, the details are to be reported elsewhere. In summary, the basic outlines of the pilot actions are as follows.

Croatia: Croatian Institute of Public Health is in collaboration with Primary Health Care Centers implementing a core set of diabetes indicators to primary care.

Finland: Finnish Institute for Health and Welfare is implementing culturally sensitive lifestyle intervention for Somalis in Finland. 

Greece: Aristotle University of Thessaloniki is implementing education and training modules for patients with hypertension and diabetes. 

Slovenia: General Hospital Novo Mesto and Primary Healthcare Centre Novo Mesto are implementing a model for integration of care across primary and secondary level of healthcare for people with complex states. 

Serbia: Faculty of Medicine, University of Belgrade, Institute of Public Health of Serbia, and Ministry of Health Republic of Serbia are implementing a network of Primary Care Diabetes Units.

Pilots using mHealth tools are being implemented in Spain (Ministry of Health Cantabria), Bulgaria (National Center of Public Health and Analyses), and Germany (University Hospital Regensburg).

## 4. Discussion

JA CHRODIS recommendations and quality criteria were developed on the basis of an extensive and inclusive process wherein experts and partners can hold different perspectives. Nevertheless, the desktop exercise needs to be tested and evaluated in real-life experience. 

In JA CHRODIS PLUS [14], development and implementation of practices based on JA CHRODIS recommendations is supported and closely studied so that the use of JA CHRODIS recommendations in real-life experiences is being validated and upgraded to the guide on how to use them efficiently. In broader terms, the partners aim to support a country’s efforts and transnational collaboration to implement JA CHRODIS recommendations and quality criteria and other outputs at the policy, system, and local/specific level. This is done to improve the quality of care for people with chronic diseases and to identify key factors across EU member states that foster health and care provision adapted to individual’s needs using mobile technology.

Based on the first period of the implementation and only preliminary intermediary results of the implementation, partners reported that JA CHRODIS recommendations and quality criteria was a useful and time-consuming tool to design a pilot action plan. However, from the first planning step, the plan was much more comprehensive without the use of the JA CHRODIS recommendations. It seems that the JA CHRODIS recommendations and quality criteria are very useful for the core leadership team, which is responsible for overseeing the whole process. The greatest added value from the use of the JA CHRODIS recommendations was seen from the perspective of meaningful patient/user involvement in co-designing the pilot actions and the perspective of post-project sustainability and scalability. Since it could be predicted that a few months after the implementation started, the energy of implementing teams would be exhausted, and so the partners agreed to put in an intermediary evaluation based on key performance indicators as defined by their pilot action plans, and intermediary self-evaluation against JA CHRODIS quality criteria. The results were shown and debated at the study visit, thus helping to develop another big-step PDSA cycle. Study visits were planned in the early phase of pilot actions also to foster the activities via knowledge and experience exchange. They were designed from a patient/person perspective to assess if pilot activities meet patients’ and/or persons’ expectations with special emphasis on the respective JA CHRODIS recommendation on empowering the target population as well as the education and training to promote empowerment. National, regional, and local stakeholders were invited together with the national partners of the joint action, thus facilitating the dissemination and communication activities and increased the potential for the sustainability and the scalability of piloted action. Most of the pilot action plans redirected their activities based on the results of the study visits in order to integrate actions that will support the sustainability of the implemented action even after the end of joint action.

## 5. Conclusions

The first experiences in the joint action show that JA CHRODIS recommendations and quality criteria was a useful but time-consuming tool used to design comprehensive pilot action plans, with a special focus on meaningful patient/person/user involvement. It also plans for the sustainability and/or scalability from the very beginning of the implementation. Properly prepared and conducted study visits were seen as a major boost for the adaptation of the planned activities in line with the concept of PDSA cycles and participation of key stakeholders at the study visit facilitated communication activities and increased the potential for the sustainability and the scalability of piloted action. 

## Figures and Tables

**Table 1:** Joint action in chronic diseases (JA CHRODIS), quality criteria, defined and ranked by RAND-modified DELPHI process.

Criteria	Criteria Weight	Categories	Category Weight
Practice design	14	The practice aims, objectives, and methods were clearly specified	19
		The design builds upon relevant data, theory, context, evidence, and previous practice including pilot studies	18
		The structure, organization, and content of the practice were defined, and established together with the target population	14
		There was a clear description of the target population (i.e., exclusion and inclusion criteria and the estimated number of participants)	13
		The practice includes an adequate estimation of the human resources, material, and budget requirements in clear relation with committed tasks	13
		There was a clear description of the target population, caregivers, and professional’s specific role	12
		In design, relevant dimensions of equity are adequately taken into consideration and are targeted (i.e., gender, socioeconomic status, ethnicity, rural-urban area, vulnerable groups)	11
			100
Target population empowerment	13	The practice actively promotes target population empowerment by using appropriate mechanisms (e.g., self-management support, shared decision-making, education-information, value clarification, active participation in the planning process, and in professional training).	50
		The practice considered all stakeholders needs in terms of enhancing/acquiring the right skills, knowledge, and behavior to promote target population empowerment (target population, caregivers, healthcare professionals, policy makers, etc.)	50
			100
Evaluation	13	The evaluation outcomes were linked to action to foster continuous learning and/or improvement and/or to reshape the practice	31
		Evaluation outcomes and monitoring were shared among relevant stakeholders	26
		Evaluation outcomes were linked to the stated goals and objectives	25
		Evaluation took into account social and economic aspects from both target population and formal and informal caregiver perspectives	18
			100
Comprehensiveness of the practice	11	The practice has considered relevant evidence on effectiveness, cost-effectiveness, quality, safety, etc.	38
		The practice has considered the main contextual indicators	33
		The practice has considered the underlying risks of the target population (i.e., validated tools to individual risk assessment)	29
			100
Education and training	11	Educational elements are included in the practice to promote the empowerment of the target population (e.g., strengthen their health literacy, self-management, stress management, etc.)	40
		Relevant professionals and experts are trained to support target population empowerment	30
		Trainers/educators are qualified in terms of knowledge, techniques, and approaches	30
			100
Ethical considerations	11	The practice is implemented equitably (i.e., proportional to needs)	25
		The practice objectives and strategy are transparent to the target population and stakeholders involved	25
		Potential burdens of the practice (i.e., psychosocial, affordability, accessibility, etc.) are addressed and there is a balance between benefit and burden	25
		Target population has right to be informed, to decide about their care, and their participation and confidentiality were respected and enhanced	25
			100
Governance	10	The practice included organizational elements, identifying the necessary actions to remove legal, managerial, and financial or skill barriers	15
		The contribution of the target population, caregivers, and professionals was appropriately planned, supported, and resourced	13
		The practice offers a model of efficient leadership	13
		The practice creates ownership among the target population and several stakeholders considering multidisciplinary, multi-/intersectoral, partnerships and alliances, if appropriate.	11
		There was a defined strategy to align staff incentives and motivation with the practice objectives	10
		The best evidence and documentation supporting the practice (guidelines, protocols, etc.) was easily available for relevant stakeholders (e.g., professionals and target populations)	10
		Multidisciplinary approach for practices is supported by the appropriate stakeholders (e.g., professionals associations, institutions, etc.)	10
		The practice is supported by different information and communication technologies (e.g., medical record system, dedicated software supporting the implementation of screening, social media, etc.)	10
		There was a defined policy to ensure acceptability of information technologies among users (professionals and target population) i.e., enable their involvement in the process of change	8
			100
Interaction with regular and relevant systems	10	The practice was integrated or fully interacting with the regular healthcare and/or further relevant systems	42
		The practice enables effective linkages across all relevant decision makers and stakeholders	30
		The practice enhances and supports the target populations ability to effectively interact with the regular relevant systems	28
			100
Sustainability and scalability	8	The continuation of the practice has been ensured through institutional anchoring and/or ownership by the relevant stakeholders or communities	32
		The sustainability strategy considered a range of contextual factors (e.g., health and social policies, innovation, cultural trends, general economy, and epidemiological trends)	28
		There is broad support for the practice amongst those who implemented it	20
		Potential impact on the population targeted (if scaled up) is assessed.	20
Total	100		100
